# Bifidobacteria and Their Molecular Communication with the Immune System

**DOI:** 10.3389/fmicb.2017.02345

**Published:** 2017-12-04

**Authors:** Lorena Ruiz, Susana Delgado, Patricia Ruas-Madiedo, Borja Sánchez, Abelardo Margolles

**Affiliations:** Dairy Research Institute, Spanish National Research Council (Instituto de Productos Lácteos de Asturias – CSIC), Villaviciosa, Spain

**Keywords:** bifidobacteria, *Bifidobacterium*, microbiota, immunomodulation, T cell response, PRRs, MAMPs

## Abstract

*Bifidobacterium* represents a genus within the phylum Actinobacteria which is one of the major phyla in the healthy intestinal tract of humans. *Bifidobacterium* is one of the most abundant genera in adults, but its predominance is even more pronounced in infants, especially during lactation, when they can constitute the majority of the total bacterial population. They are one of the pioneering colonizers of the early gut microbiota, and they are known to play important roles in the metabolism of dietary components, otherwise indigestible in the upper parts of the intestine, and in the maturation of the immune system. Bifidobacteria have been shown to interact with human immune cells and to modulate specific pathways, involving innate and adaptive immune processes. In this mini-review, we provide an overview of the current knowledge on the immunomodulatory properties of bifidobacteria and the mechanisms and molecular players underlying these processes, focusing on the corresponding implications for human health. We deal with *in vitro* models suitable for studying strain-specific immunomodulatory activities. These include peripheral blood mononuclear cells and T cell-mediated immune responses, both effector and regulatory cell responses, as well as the modulation of the phenotype of dendritic cells, among others. Furthermore, preclinical studies, mainly germ-free, gnotobiotic, and conventional murine models, and human clinical trials, are also discussed. Finally, we highlight evidence supporting the immunomodulatory effects of bifidobacterial molecules (proteins and peptides, exopolysaccharides, metabolites, and DNA), as well as the role of bifidobacterial metabolism in maintaining immune homeostasis through cross-feeding mechanisms.

## Early Colonization of Bifidobacteria and Proper Immune Development

Microbiota establishment in newborns involves the assembly of a novel microbial community, a process that is dependent on several factors, including the mother’s physiology (age, metabolic state, lifestyle, or even the potential transfer of microorganisms from mother to child before birth), mode of delivery, genetic background, environmental factors, type of feeding and early antibiotic use, among others (Hill et al., 2017). Similar results were found for preterm neonates, which are less abundantly colonized by bifidobacteria (Arboleya et al., 2012). Infant feeding is also a critical factor for bifidobacterial establishment in the gut, and breast-fed infants have been shown to possess higher levels of bifidobacteria than formula-fed infants (Yatsunenko et al., 2012); these high bifidobacterial levels decrease after breast milk cessation (Davis et al., 2016).

Pioneering studies revealed reduced levels of bifidobacteria in the gut microbiota composition of infants at high risk of atopic disease at 3 weeks and 3 months of age, and a higher incidence of atopic disease was found in this group of infants by the age of 1 year (Kalliomaki et al., 2001). Similarly, lower bifidobacterial levels were found in 3-month-old infants who later developed atopy at 2 years of age, or asthma at 4 years of age (Fujimura et al., 2016). All these data point to a critical role for bifidobacteria in the maturation of our immune system from gestation to childhood, suggesting that the low abundance of these early colonizers is associated with a deviated physiological state in infancy. Indeed, current evidence suggests a role of early life bifidobacteria establishment in programming future health. Therefore, it is of great importance to know the specific strains (and species) able to regulate immune responses, either directly or indirectly through the modulation of the gut microbiota, and the underlying mechanisms, in order to design dietary strategies focused on preventing immune-related disorders.

## Strain-Specific Immunomodulatory Activities/*In Vitro* and *In Vivo* Models of Study

### *In Vitro* Models

**FIGURE 1:**
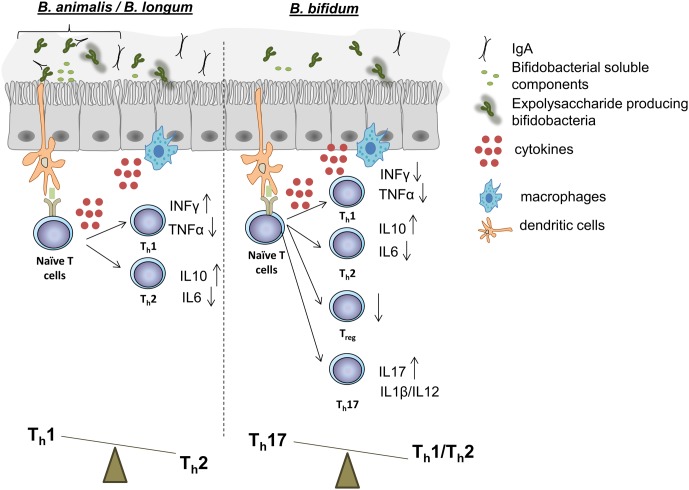
Schematic representation of the effects on immune functions that certain strains of *B. animalis*, *B. longum* **(Left)**, and *B. bifidum* **(Right)** have demonstrated in *in vitro* and *in vivo* experiments. Many *B. animalis* and *B. longum* strains have demonstrated capacity to promote a Th1 response, while, on the contrary, some *B. bifidum* strains have been revealed capable to induce a Th17 polarization. Treg responses can also be regulated by certain strains of other species. Immunomodulatory properties are strain-dependent, and further evidence is needed in order to give to each bifidobacteria species a specific immune response in the intestinal mucosa.

*In vitro* models have important limitations but they enable the preliminary screening of the effects that bacterial cells or fractions might have on different components of the immune response (Kobayashi et al., 2017). Most *in vitro* models based on immune cells employ peripheral blood mononuclear cells (PBMCs). In this way, whole cells of *B. longum*, *B. breve*, *B. bifidum*, and *B. animalis* subsp. *lactis* strains demonstrated capacity to induce dendritic cell (DC) maturation, and a species/strain-dependent T cell polarization response (Medina et al., 2007; López et al., 2010; Nicola et al., 2016). These studies revealed that, while *B. animalis* and many *B. longum* strains induced the production of the modulatory cytokine IL10 to varying degrees, the greatest strain-dependent differences were displayed in TNFα and INFγ production (**Figure 1**). Stimulation of PBMCs with subcellular fractions of bifidobacteria, including cytoplasmic, surface extracts, and supernatants, has also allowed the identification of molecular determinants of the elicited effects. For instance, a trypsin-labile cytoplasmic fraction of a *B. bifidum* strain was identified as the effector of CD8^+^ T cell activation; and supernatants of *B. breve* BB99 and *B. longum* 1941 exerted a regulatory T cell induction (Mouni et al., 2009). PBMC models are thus useful to identify desirable immune profiles in probiotic strain screenings (Liu et al., 2016).

Other *in vitro* models differentiate DCs, a specialized type of antigen presenting cells, from monocytes. DCs are regarded as the main guardians of the intestinal mucosa and are important in initiating the microbiota–immune system cross-talk. Their pattern recognition receptors (PRRs) interact with specific microbial-associated molecular patterns (MAMPs), which orchestrated molecular cascades that will determine the nature of the immune response (Hoarau et al., 2008; Wittmann et al., 2013). *In vitro* differentiated DCs allowed the identification of specific domains of a *B. bifidum* surface protein and the exopolysaccharide (EPS) of *B. longum* 35624, as the effectors of the immune responses elicited by the strains (Guglielmetti et al., 2014; Schiavi et al., 2016). DC models have also been used to predict the anti-inflammatory potential of bifidobacterial strains/molecules in specific population groups; for instance, bifidobacteria improved antigen uptake and processing by DC from Crohn’s disease patients (Strisciuglio et al., 2015).

Other *in vitro* models using immune cells employ murine splenocytes (Tanabe et al., 2008; Srutkova et al., 2015), macrophage-like cell lines (He et al., 2002; Lee et al., 2012; Mokrozub et al., 2015), or cells isolated from the gut-associated lymphoid tissues (GALT) (Hidalgo-Cantabrana et al., 2014), although they have not been widely used to examine the immunomodulation potential of bifidobacteria and thus their utility to predict immune responses is yet to be confirmed.

The immunomodulation potential of bifidobacteria has also been studied on enterocytes including Caco-2 or HT29 cell models (Bahrami et al., 2011; Chichlowski et al., 2012; Khokhlova et al., 2012; Arboleya et al., 2015; Sánchez et al., 2015; Luongo et al., 2017). Although the immune response of epithelial cells is much more limited than the one exerted by specialized immune cells, enterocytes are more directly exposed to the intestinal milieu and are considered to play a key role in initiating the bifidobacteria–host interactions.

Beyond that, co-culture systems employing both immune and intestinal cells have also been implemented to study microbial–host interactions and promise to overcome some of the limitations of single cell type models (Duell et al., 2011). However, few studies have used them on bifidobacteria (Pozo-Rubio et al., 2011). The application of organized multicellular systems like intestinal organoids for these kinds of studies is envisaged (Noel et al., 2017).

### *In Vivo* Models

Germ-free (GF) and conventional *in vivo* models, including healthy and disease-induced models, have shed light on the immune modulation capability of bifidobacterial strains including live and heat-killed cells (Sugahara et al., 2017). Screening of a large collection of gut symbionts on GF mice identified a *B. adolescentis* strain which induces a robust Th17 response, albeit not inducing intestinal inflammation (Tan et al., 2016). However, immune responses may vary strongly depending on the health status of the host, as the human sera of *Clostridium difficile* patients were shown to be more reactive against *B. longum* extracts than that of healthy individuals (Górska et al., 2016).

*In vivo* models of intestinal diseases have demonstrated the potential of *B. bifidum* and *B. animalis* strains to restore immune markers and intestinal barrier in low chronic inflammation models (Philippe et al., 2011). Similarly, *B. longum* CECT 7347 attenuated the production of inflammatory cytokines and the CD4^+^ T cell-mediated immune response in a gliadin-induced enteropathy model (Laparra et al., 2012). Other disease models, described below, have been tested in literature. In food-allergy models, a vesicle-derived protein from *B. longum* (Kim et al., 2016) and a *B. animalis* (Ezendam et al., 2008) strain, administered during lactation, exerted immunomodulatory effects. In a gut model, *B. longum* strain 51-A reduced inflammation (Vieira et al., 2015). Finally, in obesity models, *B. pseudocatenulatum* restored the lymphocyte–macrophage balance and *B. adolescentis* IM38 improved high-fat-diet induced colitis inhibiting NF-κB activation (Moya-Pérez et al., 2015; Lim and Kim, 2017). Furthermore, the role of bifidobacteria in responsiveness to immunotherapy has recently been suggested. Accordingly, using tumor models in mice, *Bifidobacterium* administration was shown to improve tumor-specific immunity and response to therapy through augmented DC function, opening new avenues to exploit the bifidobacterial-immune dialog in the context of this disease (Sivan et al., 2015).

Finally, non-murine *in vivo* models, like pig models, are very attractive for the study of microbe–host interactions due to the similarities in the gastrointestinal function and development between pigs and humans. In this context, bifidobacterial administration in neonatal piglets has been shown to increase the production of intestinal IL-10 (Herfel et al., 2013), and to improve B and T cell responses following rotavirus vaccination (Vlasova et al., 2013; Kandasamy et al., 2014; Ishizuka et al., 2016). In addition, colonization with a combination of lactobacilli and bifidobacteria in non-vaccinated gnotobiotic piglets reduced the severity of rotavirus infection, while in vaccinated animals enhanced Th1 (Chattha et al., 2013). Thus, *in vivo* models closer to humans are valuable to study the immunomodulatory potential of certain strains, should be “particularly in the context of pig models in order to study pre-term birth and necrotizing enterocolitis (NEC)” (Oosterloo et al., 2014).

### Humans

Different immunoreactive proteins from two *B. longum* strains have been identified in mono-colonized mice, rabbit, and human sera, revealing that the effects are strain and host specific (Górska et al., 2016) and emphasizing the need to further support *in vitro* immunomodulatory effects in clinical trials. A summary of human studies that focus on the immunomodulatory effects of bifidobacterial consumption in multiple disorders, in some of which gut microbial ecology dysbiosis and altered immune profiles coexist, is presented in **Table 1**.

**Table 1:** *Bifidobacterium* role on diseases with an immunological component.

Species/strains tested	Population	Observations	Reference
**Intervention studies**			
*B. lactis* BB12	Healthy adults	Four weeks administration of yogurt with the strain resulted in lower expression of TLR-2 on CD14^+^HLA-DR^+^ cells and reduction in TNF-α secretion	Meng et al., 2017
*B. lactis* Bi-07	Healthy elderly adults	Four weeks administration improved phagocytic activity of monocytes and granulocytes	Maneerat et al., 2014
*B. animalis* ssp. *lactis* HN019	Systematic meta-analysis on four clinical trials/healthy elderly subjects	The strain supplementation resulted in increased PMN phagocytic capacity and moderately increased NK cell tumoricidal activity	Miller et al., 2017
*B. animalis* LKM512	Atopic dermatitis adult patients	Administration into a yogurt daily for 4 weeks induced a Th1-type cytokine profile	Matsumoto et al., 2007
*B. lactis* NCC2818	Seasonal allergic rhinitis to grass pollen/adults	Eight weeks probiotic administration reduced Th2-cytokines secretion and CD63 expressing basophiles correlating to improved symptoms	Singh et al., 2013
*B. lactis* HN019	Metabolic syndrome patients	Decrease in TNFα and IL6 correlated to improvement in cardiovascular risk markers	Bernini et al., 2016
*B. breve* BR03 and *B. breve* B632	Cystic fibrosis/children	Three months administration of the two strains combination reduced proinflammatory markers	Klemenak et al., 2015
*Lb. gasseri KS-13*, *B. bifidum G9-1*, and *B. longum MM-2*	Healthy elderly population	Three weeks administration of probiotic mix maintained CD4+ lymphocytes and resulted in a more anti-inflammatory cytokines profile with increased IL-10	Spaiser et al., 2015
*B. breve* M-16V and *B. longum* BB536	Prenatal administration to pregnant mothers 1 month prior delivery and to the infants during 6 months	Reduced risk of developing eczema in the probiotic group	Enomoto et al., 2014
*B. longum* BB536, *B. infantis* M-63, *B. breve* M-16V mixture	Seasonal allergic rhinitis and intermittent asthma/children	Improvement of symptoms following 4 weeks of probiotic administration	Miraglia Del Giudice et al., 2017
*B. longum* BB536	Healthy newborns	The number of IFN-γ secretion cells and the ratio of IFN-γ/IL-4 secretion cells was increased, suggesting improvement of Th1 function	Wu et al., 2016
*B. longum* BB536	Elderly subjects receiving enteral tube feeding	Twelve weeks administration resulted in increased serum IgA and maintenance of NC cell activity	Akatsu et al., 2013
*B. longum* 35624	Patients of ulcerative colitis (UC), chronic fatigue syndrome (CFS), and psoriasis, as compared to healthy controls	Six to eight weeks of probiotic administration reduced CRP, TNFα, and IL6 in UC, CFS, and psoriasis patients	Groeger et al., 2013
*B. infantis* NLS	Celiac adults	Six weeks probiotic administration reduced Paneth cells numbers and expression of α-defensine-5, as compared to patients under a gluten-free diet without probiotic supplementation	Pinto-Sánchez et al., 2017
*B. longum* CECT 7347	Children with newly diagnosed coeliac disease	Three months administration resulted in reduced peripheral CD3+ lymphocytes, TNFα, and sIgA in stools	Olivares et al., 2014
**Observational studies**			
*B. breve*	Eczema risk in children at high risk of allergic disease	Early *B. breve* colonization was associated to reduced risk of eczema	Ismail et al., 2016
*Bifidobacterium* spp. and *B. adolescentis*	Allergic asthma in adults	Reduction in gut bifidobacterial representation and *B. adolescentis* prevalence within the bifidobacterial group in the studied population	Hevia et al., 2016
*B. pseudocatenulatum*	Gout patients	*B. pseudocatenulatum* depletion in gout patients	Guo et al., 2016

*Summary of observational and intervention studies in humans*.

## Molecular Structures Driving Specific Immunomodulatory Effects

Findings from the last 10 years support the idea that bifidobacteria exert their beneficial effects on host health through the immunomodulatory action of some of their surface-associated molecules (Hoarau et al., 2006; Ewaschuk et al., 2008). This is based on the interaction of a specific bifidobacteria molecule, a MAMP, with a PRR presents on the membrane of epithelial/immune cells, which mostly configures the cellular structure of the intestinal mucosa (Sutterwala and Flavell, 2009). Although mucosa itself is differently organized, depending on the gut section considered, bifidobacteria are thought to exert their immunomodulatory activity mainly in the colon and in the distal part of the ileum, where up to 46% of the Peyer’s patches are located (Van Kruiningen et al., 2002). Scientific evidence has shown the presence of immunomodulatory compounds in bifidobacteria spent medium which are released during bacterial growth (**Figure 1**).

### Proteins and Peptides

Bifidobacterial proteins are one of the targets of human immunoglobulins, notably IgA, which is secreted into the gut lumen in order to control the commensal microbiota populations. Up to six different extracellular proteins from the strains *B. longum* subsp. *longum* NCIMB 8809, *B. bifidum* LMG 11041^T^, and *B. animalis* subsp. *lactis* IPLA 4549 were recognized by pooled sera from healthy individuals, or Inflammatory Bowel Disease (IBD) patients (Hevia et al., 2014). Perhaps the best known example of an immunomodulatory protein is the extracellular serpin secreted by *B. longum* subsp. *longum*. Serpin stands for serine protease inhibitor and includes different families that share the ability to bind and irreversibly inactivate proteases. The gene coding for serpin is not widely distributed among the genus *Bifidobacterium*, being present in up to nine species so far (Turroni et al., 2010a). More precisely, the targets of serpin secreted by *B. longum* are two important pro-inflammatory proteases: human neutrophil and pancreatic elastases (Ivanov et al., 2006), proteases that have been shown to induce the serpin gene through a two-component regulatory system (Alvarez-Martin et al., 2012). Limiting the local action of these proteases suggests a role of bifidobacteria in the maintenance of gut homeostasis.

Other well-known protein structures with an immunomodulatory action are pili, which self-assemble on the bifidobacteria surface in the form of filaments and have a primary function of adherence to the intestinal surface (Turroni et al., 2014). Lower levels of IL10 and higher levels of TNFα were detected in the murine cecum mucosa as a response to the presence of a *Lactococcus lactis* strain, genetically modified for producing *B. bifidum* pili. This response was not observed in the wild-type strain, suggesting a specific interaction of these structures with the gastrointestinal mucosa (Turroni et al., 2013). Another protein with an immunomodulatory effect is the peptidoglycan hydrolase TgaA, a surface-associated protein in *B. bifidum*, which was shown to induce IL2 production in monocyte-derived dendritic cell (MoDC), the key cytokine in T_reg_ cell expansion (Zelante et al., 2012; Guglielmetti et al., 2014). Finally, our own work has revealed the presence of immunomodulatory peptides encrypted in the sequences of bifidobacteria proteins. In this sense, a peptide contained within the sequence of the protein translocase subunit SecA of *B. longum* DJ010A triggered a marked Th17 response when incubated with human PBMCs (Hidalgo-Cantabrana et al., 2017b).

### EPSs

EPSs are carbohydrate polymers that are synthesized and exhibited in the bifidobacterial surface (Hidalgo-Cantabrana et al., 2014). Although the exact molecular mechanisms have not been described so far, EPSs have a great impact on the host immune function (Hidalgo-cantabrana et al., 2012). In a murine model, the EPS-producing strain *B. breve* UCC2003 was associated with increases in the mucosal levels of the pro-inflammatory IL12, INFγ, and TNFα which turned out to protect against *Citrobacter* infection (Fanning et al., 2012). Murine J77A.1 macrophages challenged with the EPS produced by strain *B. longum* BCRC 14634 increased the production of the anti-inflammatory cytokine IL10 when compared to basal conditions, and when challenged with lipopolysaccharide, the presence of the EPS was linked to lower levels of the pro-inflammatory cytokine TNFα (Wu et al., 2010). It is noteworthy that the rhamnose-rich, high-molecular weight EPS isolated from the strain *B. animalis* subsp. *lactis* IPLA-R1 was able to increase IL10 production in a PBMC model and to decrease the TNFα production in human colonic biopsies (Hidalgo-Cantabrana et al., 2015). Moreover, the administration of strain IPLA-R1 to Wistar rats was associated with higher serum levels of TGFβ and lower serum levels of the pro-inflammatory interleukin IL6 (Salazar et al., 2014).

EPS produced by specific bifidobacteria strains have been shown as molecules able to prevent exacerbated pro-inflammatory responses. *B. longum* subsp. *longum* 35624 is a strain which has shown clinical efficacy in Irritable Bowel Syndrome, a human condition cursing with chronic mucosal inflammation (Altmann et al., 2016). The anti-inflammatory effects elicited by this strain were shown to rely in its surface-associated EPS, which prevented expansion of the pro-inflammatory Th17 response compared to an exopolysaccharide-negative mutant derivative (Schiavi et al., 2016).

Finally, recent data on a mouse model of pathological cell shedding, EPS from *B. breve* UCC2003 appeared to confer protective effect through MyD88-dependent signaling (Hughes et al., 2017). Diversity of gene clusters responsible for EPS biosynthesis is high among bifidobacterial species/strains (not to mention variations in the level of EPS production) and this diversity may hold tremendous potential for strain-specific immune responses.

### DNA

Bifidobacteria possess genomes with high G+C proportions, and un-methylated CpG motifs derived from them can interact with the TLR 9 present on immune cells. Several publications have reported on the immunomodulatory activity of bifidobacterial DNA. CpG motifs have in one case been linked to a promotion of the T_h_1 response, dedicated to fight intracellular pathogens such as viruses (Ménard et al., 2010). Another work described an oligodeoxynucleotide derived from the *B. longum* BB536 strain able to inhibit anti-ovalbumin–IgE titres in a murine model of type-I allergic response after ovalbumin injection (Takahashi et al., 2006).

## Bifidobacterial Metabolism Triggers Cross-Feeding Mechanisms that Maintain Immune Homeostasis in the Gut

Many efforts are currently being pursued to understand the metabolic fluxes within the gut ecosystem among bifidobacteria, other members of the gut microbiota and the human host (Hidalgo-Cantabrana et al., 2017a). A major metabolic contribution elicited by bifidobacteria from their host is represented by the breakdown of non-digestible, diet-derived glycans, and carbohydrates provided by the host known as host-derived glycans [mucins and human milk oligosaccharides (HMOs)] (Milani et al., 2015). Mucin is a host-produced glycan that constitutes one of the main barriers covering the gastrointestinal mucosa (Tailford et al., 2015). Among bifidobacteria, only members of *B. bifidum* species have been shown to efficiently metabolize mucin (Ruas-Madiedo et al., 2008; Turroni et al., 2010b; Ruiz et al., 2011). HMOs are present in high concentrations in human colostrum and breast milk. Bifidobacteria, which dominate during early life, are among the best described gut bacteria with the ability to utilize HMOs. Several species possess glycosyl hydrolases that cleave specific linkages within the HMO molecules, the best characterized being those synthesized by *B. bifidum* (Ruiz et al., 2016). HMOs are preferentially fermented by *B. bifidum* and *B. longum* species which, together with *B. breve*, are the most abundant in breast-fed infant gut microbiota (Sela and Mills, 2010). Thus, the ability of these species to utilize these otherwise indigestible carbohydrates explains their abundance in breast-fed neonates (Zivkovic et al., 2011).

Metabolic cross-feeding mechanisms in the gut are commonly exploited by primary microbial degraders like bifidobacteria which, thanks to partial extracellular hydrolysis of specific complex carbohydrates (e.g., host-produced glycans), provide monosaccharides, oligosaccharides, or metabolites for other microbial gut inhabitants (De Vuyst and Leroy, 2011). As an example, *B. bifidum* PRL2010 is a strain specialized in the extracellular breakdown of host-glycans and, thus, in the release of simple sugars that can be utilized by other members of the (bifido)bacterial community (Turroni et al., 2016). The subsequent fermentative metabolism of these carbohydrates generates end-metabolites, such as acetate and lactate, which are the main end-products of the bifidobacteria catabolism. Acetate released in the gut by bifidobacteria is used as substrates for other microbial gut fermenters, mainly butyrate and propionate producers (Flint et al., 2015). The production of these two major short-chain fatty acid metabolites have been shown to have anti-inflammatory effects, and promotes and regulates the pool of colonic T_reg_ cells (Arpaia et al., 2013; Smith et al., 2013). By inhibiting histone deacetylase activity in DC and T cells, butyrate acts in the differentiation of T_reg_ cells, increasing the expression of the T_reg_ marker FoxP3 (Furusawa et al., 2013). Signalization has been proposed to be mediated by the butyrate receptors in epithelial and immune cells named FFAR3 (free fatty acid receptor 3) and GPR109A (Ahmed et al., 2009; Remely et al., 2014).

## Concluding Remarks

Bifidobacterial cells, their subcellular fractions, or specific molecules produced by these microorganisms, hold an important potential to trigger immunomodulatory responses involved in the maintenance of our healthy physiological state. However, these responses are poorly understood and need for more research on how this molecular communication between bifidobacteria and host cells is performed. Additionally, the increasing knowledge on the role played by different gut microbiota members, and the understanding of the cross-talk and cross-feeding interaction processes between bifidobacteria, the host, and the surrounding network of intestinal microbes, should facilitate the synergistic use of different intestinal microorganisms to modulate the immunological and inflammatory processes in a microbial dependent way.

## Author Contributions

AM and BS designed the structure of the mini-review. LR, SD, PR-M, BS, and AM wrote the manuscript and drafted the first version of the manuscript. All authors reviewed the final version of the manuscript.

## Conflict of Interest Statement

The authors declare that the research was conducted in the absence of any commercial or financial relationships that could be construed as a potential conflict of interest.
